# Measuring Coverage in MNCH: Evaluation of Community-Based Treatment of Childhood Illnesses through Household Surveys

**DOI:** 10.1371/journal.pmed.1001384

**Published:** 2013-05-07

**Authors:** Elizabeth Hazel, Jennifer Requejo, Julia David, Jennifer Bryce

**Affiliations:** Institute for International Programs, Johns Hopkins Bloomberg School of Public Health, Baltimore, Maryland, United States of America; Professor of Demography and Social Statistics, University of Southampton, United Kingdom

## Abstract

Community case management (CCM) is a strategy for training and supporting workers at the community level to provide treatment for the three major childhood diseases—diarrhea, fever (indicative of malaria), and pneumonia—as a complement to facility-based care. Many low- and middle-income countries are now implementing CCM and need to evaluate whether adoption of the strategy is associated with increases in treatment coverage. In this review, we assess the extent to which large-scale, national household surveys can serve as sources of baseline data for evaluating trends in community-based treatment coverage for childhood illnesses. Our examination of the questionnaires used in Demographic and Health Surveys (DHS) and Multiple Indicator Cluster Surveys (MICS) conducted between 2005 and 2010 in five sub-Saharan African countries shows that questions on care seeking that included a locally adapted option for a community-based provider were present in all the DHS surveys and in some MICS surveys. Most of the surveys also assessed whether appropriate treatments were available, but only one survey collected information on the place of treatment for all three illnesses. This absence of baseline data on treatment source in household surveys will limit efforts to evaluate the effects of the introduction of CCM strategies in the study countries. We recommend alternative analysis plans for assessing CCM programs using household survey data that depend on baseline data availability and on the timing of CCM policy implementation.

*This paper is part of the* PLOS Medicine “*Measuring Coverage in MNCH” Collection*

## Introduction

Most low- and middle-income countries are making slow progress in addressing child mortality—too slow to achieve Millennium Development Goal 4 by 2015 [1]. Diarrhea, pneumonia, and malaria account for 37% of under-five deaths worldwide [2], with only about one-third of children with these illnesses receiving appropriate treatment [3]. To address this disease burden and treatment gap, governments and donors in 52 of the Countdown to 2015 priority countries had adopted community case management (CCM) of childhood illness strategies by 2011 [1],[4]. CCM aims to extend the treatment of childhood illnesses from health facilities into communities [5]–[7] by training and supporting existing or newly recruited community health workers to provide treatment for neonatal conditions and simple cases of childhood pneumonia, diarrhea, and malaria at the community level and to refer cases of more severe illness. The underlying assumption of CCM is that the expansion of treatment capabilities to community health workers will result in increases in access to and coverage of treatment, especially for children living in households far removed from existing health facilities [6].

Clearly, it is essential that countries introducing CCM carefully assess its contribution to increased treatment coverage for childhood illnesses. To do this, population-level data on the place of treatment and the type of health provider are needed. Where these data exist, they can serve as a baseline for evaluations of the contribution of CCM to treatment coverage going forward. Where they do not exist, evaluators will need alternative analytical designs. In practice, routine health information systems in low- and middle-income countries are often weak and cannot consistently provide valid coverage data for these treatment indicators. Often the best available source of coverage data in these countries is nationally representative household surveys [8]. Two major programs generate the household-level survey data needed to measure coverage for maternal, newborn, and child health interventions in low- and middle-income countries—the Demographic and Health Surveys (DHS), supported by USAID [9], and the Multiple Indicator Cluster Surveys (MICS), supported by UNICEF [10]. However, although questions on the coverage of care seeking and appropriate treatment of childhood illnesses have been included in DHS and MICS protocols in the past, it may not be possible to use these data to determine whether a treatment was delivered by a health facility or by a community-based health worker, information that is needed to assess the success of CCM.

In this review, which is part of the *PLOS Medicine* “Measuring Coverage in MNCH” Collection, we assess the extent to which existing household surveys provide the data needed to measure trends in coverage for the correct management of childhood illnesses by place of treatment (health facility or community) and type of provider. In addition, we recommend alternative analysis plans that might be used in settings where baseline data are insufficient to measure trends in treatment coverage by the place and type of provider.

## Assessing the Surveys

For our assessment, we focused on Ethiopia, Ghana, Malawi, Mali, and Niger, five countries where the Catalytic Initiative to Save a Million Lives, a partnership of donors and United Nations agencies, is supporting CCM as a strategy to accelerate coverage for the treatment of childhood illnesses [11]. In total, we reviewed the locally adapted questionnaires from nine DHS and MICS surveys conducted in these countries since 2005 along with the most recent DHS and MICS core questionnaires.

We assessed each survey to determine whether it could provide information on the place of treatment—in a health facility or in the community—for a child reported to have symptoms of pneumonia, fever, and/or diarrhea, and information about the provider of the treatment. We defined symptoms of pneumonia as cough and rapid or difficult breathing, although DHS refers to these symptoms as “symptoms of acute respiratory infection” and MICS refers to them as indicating “suspected pneumonia.” Fever is used as a symptom of malaria in CMM strategies. We reviewed question wording, question placement, skip patterns, and the sample surveyed for each survey questionnaire. Table S1 shows all the information on place of treatment and care seeking for these three major childhood illnesses collected by the surveys in each country.

We assumed that CCM provided no treatments in the study countries as a part of the formal health system prior to the adoption of illness-specific CCM policies. Table 1 shows a summary of national CCM policies, the cadre of the CCM worker, and the date of reported CCM policy implementation for the five countries. We show the date of national policy implementation rather than policy adoption, because procurement issues often prevented full CCM implementation at the time of adoption of the policy (for example, treatment of diarrhea with zinc often lagged behind treatment with oral replacement salts). We examined the locally adapted questionnaire for each survey to assess whether the data set could be used to report on three constructs (care seeking, appropriate treatment, and place of treatment) for each of three childhood illnesses (pneumonia, diarrhea, and fever). We assigned each survey a score of “yes” or “no” for each of the three constructs and illnesses. We assigned a “partial” score if some information was available but was not sufficient to determine the exact source of care or treatment.

**Table 1:** Summary of national CCM policies, cadre of worker, and date of policy implementation for the five countries.

Country	Cadre of CCM Worker	Child Illness	1^st^-Line Treatment	Date of CCM Policy Implementation
Ethiopia	Health extension worker	Diarrhea	ORS	2004 [18]
			Zinc	2012^a^
		Pneumonia	Cotrimoxazole	2011 [19]
		Fever	Artemether/lumefantrine/Chloroquine	2004 [18]
Ghana	Community-based agent	Diarrhea	ORS	2004 [20]
			Zinc	2010 [19]
		Pneumonia	Amoxicillin	2010 [19]
		Fever	Chloroquine	2003 [20]
			ACT	2007 [20]
Malawi	Health surveillance assistants	Diarrhea	ORS	2008 [19]
			Zinc	2010^b^
		Pneumonia	Cotrimoxazole	2008 [19]
		Fever	Coartem	2008 [19]
Mali	*Agents de santé communautaires*	Diarrhea	ORS	2010 [19]
			Zinc	2010 [19]
		Pneumonia	Amoxicillin^c^	2010 [19]
		Fever	ACT	2010 [19]
Niger	*Agents de santé communautaires*	Diarrhea	ORS	2006 [21]
			Zinc	2008 [21]
		Pneumonia	Cotrimoxazole	2008 [21]
		Fever	ACT	2008 [21]

a Personal communication, Tedbabe Degefie.

b Personal communication, Humphreys Nsona.

c Personal communication, Hamadoun Sangho.

ACT, artemisinin combination therapy; ORS, oral replacement salts.

Care seeking refers to reports by caregivers about whether, and if so where, they took the sick child for care. We assigned a “yes” score to a survey for care seeking if there were specific questions about where or from whom the mother or caregiver sought advice or treatment that allowed us to determine whether care was sought from a health facility or at the community level.

We defined “appropriate” treatment as the first-line treatment recommended by the CCM policy in each country (Table 1). We assigned a “yes” score to a survey for appropriate treatment only if the survey included specific questions on treatment options. Appropriate treatments included oral replacement salts and/or zinc for diarrhea (depending upon when countries implemented policies for zinc), antibiotics for pneumonia, and specific antimalarials (sulfadoxine and pyrimethamine, artemisinin-combination therapies, etc.) for fever.

Place of treatment refers to the location (health facility or community) where the treatment was “received,” as reported by the child's mother or caregiver. In this context, “received” may mean that the mother or caregiver received either the actual medicine or a prescription for the medicine to be filled elsewhere. Response options to these questions varied across the surveys, but generally included both public and private health facilities, pharmacies and drug venders/shops, and, sometimes, specific community-based providers such as community health workers and/or traditional healers. We assigned a “yes” score for place of treatment if we were able to determine unambiguously whether the child received the treatment at a health facility or at the community level. We noted whether information was available to determine the specific community site of treatment (e.g., mobile clinic) and the specific community treatment provider (e.g., community health workers).

In addition, for surveys that did not include information on the place of treatment, we were sometimes able to draw plausible inferences about this construct based on information about care seeking or intention to treat and appropriate treatment. Thus, if the country policy at the time of the survey allowed treatment by community health workers (as opposed to referral to a health facility), it may be possible to assume that children reported as having been taken to a community health worker received treatment for their illness from that individual. Moreover, for surveys without direct questions on place of treatment, inferences based on care seeking can be strengthened if data are available on appropriate treatment. In other words, in a context where government policies support CCM, a mother who reports seeking care for her child from a community health worker *and* who reports receiving appropriate treatment can be assumed to have benefitted from CCM with more certainty than a mother who reports only that she sought care from a community health worker.

## Do Household Surveys Contain Baseline Data on CCM?

Table 2 summarizes the availability of information on care seeking, treatment, and point-of-treatment for each of the three childhood illnesses in the large-scale household surveys conducted in the five countries that we studied. We grouped the results by type of survey (DHS versus MICS) to reduce redundancy.

**Table 2:** Information on care seeking, treatment, and point-of-treatment available from the large-scale household surveys for the five countries.

Country and Survey	Care Seeking:			Appropriate Treatment:		Place of Appropriate Treatment:	
	Available	CCM Health Provider Listed as Possible Response	Additional Information	Available	Additional Information	Available	Treatment Provider (CCM)
**Ethiopia 2005 DHS**	Yes for all illnesses	Community health agent	Indicates first care-seeking site	Yes for all illnesses	For **pneumonia** and **fever** illnesses: asks if drug was obtained outside the home.	No	N/A
**Niger 2006 DHS**	Yes for all illnesses	For **diarrhea** illness: *Agent santé communautaire*For **pneumonia**/**fever**: no community health worker listed	No	Yes, for **diarrhea** and **fever** only	For **fever** illness: asks if drug was obtained outside the home.	No	N/A
**Mali 2006 DHS**	Yes for all illnesses	*Agent santé communautaire*	No	Yes, for **diarrhea** and **fever** only	For **fever** illness: asks if drug was obtained outside the home.	No	N/A
**Ghana 2008 DHS**	Yes for all illnesses	Government and nongovernment fieldworker	Indicates first care-seeking site	Yes for all illnesses	For **pneumonia** and **fever** illnesses: Asks if drug was obtained outside the home.	No	N/A
**Malawi 2010 DHS**	Yes for all illnesses	Government and nongovernment health surveillance assistants	Indicates first care-seeking site	Yes for all illnesses	No	No	N/A
**Ethiopia 2011 DHS**	Yes for all illnesses	Government health post/health extension workers	Indicates first care-seeking site	Yes for all illnesses	No	No	N/A
**DHS Phase 6 Core 2008–2013**	Yes for all illnesses	Government and nongovernment fieldworker	Indicates first care-seeking site	Yes for all illnesses	No	No	N/A
**Ghana 2006 MICS**	Yes, for **pneumonia** only; Partial^a^ for **fever** only	For **pneumonia** illness: village health workerFor **fever** illness: no community health workers listed	For **fever** illness: asks if child was taken to a health facility; no specific sites are listed	Yes for all illnesses	No	Yes for all illnesses	Village health worker
**Malawi 2006 MICS**	Yes, for **pneumonia** only; Partial^a^ for **fever** only	For **pneumonia** illness: village health workerFor **fever** illness: no community health workers listed	For **fever** illness: asks if child was taken to a health facility; no specific sites are listed	Yes for all illnesses	No	Partial^a^, for **fever** only	For **fever** illness: asks about specific medicines that were given at the health facility
**Mali 2009 MICS**	Yes, for **pneumonia** and **fever** only	For **pneumonia** and **fever** illnesses: no community health workers listed	No	Yes for all illnesses	No	Partial^a^, for **fever** only	For **fever** illness: asks about specific medicines that were given at the health facility
**MICS4 Core (2009–2011)**	Yes, for **pneumonia** only; Partial^a^ for **fever** only	For **pneumonia** illness: village health worker	For **fever** illness: asks if child was taken to a health facility; no specific sites are listed	Yes for all illnesses	No	Partial^a^, for **fever** only	For **fever** illness: asks about specific medicines that were given at the health facility

a Partial indicates that some information is available but not enough to determine exact source of care or treatment.

### DHS Surveys

The Mali 2006 and Niger 2006 surveys occurred before the implementation of the national CCM policy (Table 1). Ghana (2008) and Ethiopia (2005) had implemented CCM policies at the time of the surveys for diarrhea (excluding zinc treatment) and fever, but not for pneumonia. Ethiopia had implemented the CCM policy for pneumonia by the time of the 2011 DHS. Malawi was implementing CCM policies for all three illnesses at the time of the 2010 DHS.

All the DHS surveys included specific questions on care seeking. Response options for the site of care included a mix of both community and health facility sites and providers in the public and private sectors. The surveys for Ethiopia, Ghana, Mali, and Malawi included a community health worker as a possible response option for the care-seeking questions for the three childhood illnesses reviewed. The Niger 2006 DHS listed a community health worker for diarrhea illness care seeking only.

Treatment questions were available in all the DHS surveys with the exception of pneumonia treatment in the 2006 surveys conducted in Mali and Niger. In addition, there were no questions on zinc treatment for diarrhea in these two surveys; however, these countries had not incorporated zinc into their national policies by the time of the surveys.

Four of the DHS surveys (Ghana 2008, Malawi 2010, and Ethiopia 2005 and 2011) and the phase 6 core questionnaire included a follow-up question for mothers reporting that care had been sought at more than one site that was designed to determine the site where care was sought first for each illness.

Explicit place of treatment information was not available from DHS surveys conducted since 2005 in any of five countries we considered and was also not included in the DHS phase 6 core questionnaire. However, as explained earlier, we found that information on place of treatment could be gleaned from the analysis of care-seeking data collected in DHS surveys, although the results were limited in important ways. For example, independent question sequences in the DHS surveys asked a mother about whether (and where) she sought advice or treatment for her child, and about the treatment received. Both questions offered locally adapted response options that included both facility and community sites, so one could assume that if the child received appropriate treatment, it was through the reported site or provider. Because the care-seeking question allowed for multiple responses, the sequence through which care was sought from specific sites was not captured (except in the questionnaires that include a follow-up question to identify first site of care seeking). Thus, there was no way to determine through the existing questions what treatment, if any, was given at the site(s) where care was sought, unless only one source of care seeking was mentioned, in which case one could presume (possibly incorrectly) the treatment was received there.

### MICS Surveys

Ghana had implemented CCM policies for diarrhea and fever treatment at the time of its MICS survey, but neither Mali not Malawi had implemented a national CCM policy at the time of their surveys (Table 1).

Questions about care seeking for pneumonia were included in all four MICS surveys examined here (Table 2). The response options included community health workers in the Ghana and Malawi surveys and in the core MICS4 questionnaires. Information on care seeking for diarrhea was not captured by these surveys. The Ghana and Malawi surveys as well as the MICS4 core questionnaire partially captured information on care seeking for fever through questions about whether a child with fever was taken to a health facility.

All the MICS surveys included questions about the treatments received for children reporting symptoms of pneumonia, diarrhea, or fever. The Malawi and Ghana 2006 surveys did not include questions on zinc; however, a policy recommending treatment of diarrhea with zinc had not been implemented at the time of the surveys in the two countries. Two country surveys (Malawi 2006 and Mali 2009) and the MICS4 core questionnaire asked a follow-up question for children reported to have received treatment for fever to identify which drugs were given or prescribed through a health facility, which could be used to assess the validity of responses.

Finally, Table 2 shows that information on the proportion of children treated by a community health worker was captured in the 2006 Ghana MICS survey for the three major illnesses. Partial questions on place of treatment for fever were included in the Mali (2009) and Malawi (2006) MICS surveys and in the MICS4 core questionnaire.

## An Alternative Analysis Plan for Program Implementers and Evaluators

This analysis shows that, with the exception of the 2006 Ghana MICS survey, comprehensive baseline data on the place and provider of appropriate treatment of childhood pneumonia, diarrhea, and fever are not available from the major household surveys conducted in the study countries before 2010. However, evaluators can still answer some important questions about the effectiveness of CCM in the study countries, especially if future surveys are designed to capture these data. For instance, one of the major global questions is how the introduction of CCM could affect care seeking at health facilities. Evaluators can compare survey reports of care seeking from all sources at baseline (prior to CCM program implementation) with the sum of care seeking rates from health facilities and from community-based health workers at midline (during CCM program implementation) and endline (the time of the CCM program evaluation). They can then use routine data on utilization to assess whether CCM has contributed to overall increases in treatment coverage. This approach is limited in most low- and middle-income countries due to the poor quality of routine data on service utilization [8]. Another possible approach would be to compare illness-specific care-seeking rates from health facilities at baseline with the sum of care-seeking rates from health facilities and from community-based health workers at midline and endline.

**Figure 1:**
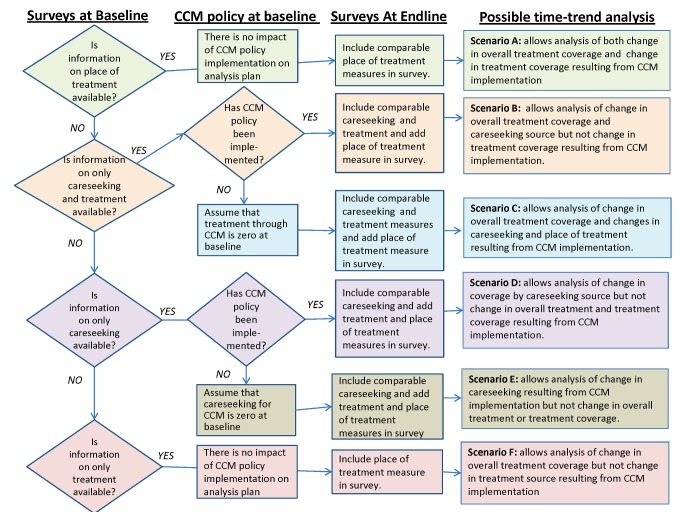
Analysis plan flow chart. Decision flow chart for six scenarios of time-trend analysis options depending on baseline data availability and timing of CCM policy implementation. CCM policies have been implemented at endline in all cases.

Figure 1 provides a flow chart of alternative analysis plans designed to determine the effects of CCM on treatment coverage, treatment source, and care-seeking source, depending on the types of baseline data that are available in a given context. Consider, for example, a hypothetical setting where a DHS or MICS survey collects information on both care seeking and treatment but not on place of treatment prior to implementation of a CCM program (baseline). After this survey is conducted, a CCM policy is implemented, and the government requests a time-trend analysis on the impact of the CCM program on childhood illnesses. If an endline survey collects comparable information on care seeking and treatment but also collects information on place of treatment, then a time-trend analysis is possible for the change in overall treatment coverage. Changes in care seeking outside health facilities can also be assessed to determine the impact of the CCM program on care-seeking patterns. Moreover, in this hypothetical setting, because it can be assumed that treatment through CCM-trained community health worker at baseline is zero, increases in community health workers as the point of treatment can be assessed (Figure 1, Scenario C).

By contrast, consider a setting where there is information only on the treatment available at baseline and not on either the place of treatment or care seeking. In this scenario, the timing of the CCM policy implementation is irrelevant since no measure of treatment or care-seeking source is collected through the baseline survey. If information on place of treatment, as well as on the actual treatment, is collected in an endline survey, then time-trend analysis is possible for the changes in overall treatment coverage that is related to the implementation of CCM but not for the change in overall treatment source (Figure 1, Scenario F).

Importantly, given that the CCM policy for the three major childhood illnesses may be implemented at different times and the variation in the availability of care seeking, treatment and place of treatment data by illness, evaluators and program implementers may need to investigate alternative illness-specific analyses.

## Limitations in Measuring the Source of Treatment through Household Surveys

The sample sizes needed to determine whether changes in treatment rates for specific childhood illnesses are statistically significant will vary depending on the prevalence of each disease (or its presumed prevalence based on respondents' reports of signs and symptoms) and estimated levels of appropriate treatment at baseline. Increasing the sample sizes will increase the costs and logistical challenges of capturing the information through a DHS/MICS survey, as discussed in another paper in the Collection [12], as will looking at place of treatment by wealth status to determine whether CCM and other service delivery strategies designed to reach the poor are effective in reducing inequities. Survey planners will need to consider whether the results obtained from such analyses will justify the resources required, and evaluators of CCM will need to interpret inadequately powered analyses with care.

There are other important limitations in using household surveys to measure appropriate treatment of childhood illnesses [13]–[15]. For example, the analyses rely on respondents' ability to recognize, recall, and report signs and symptoms correctly and to be able to accurately recall and report care-seeking patterns, where treatments were obtained, and when/how often they were given to the child. Work is underway to improve these measurements, as reflected by the other papers in this Collection. Currently, DHS and MICS surveys can provide only limited information on whether the child was appropriately assessed and on whether adequate treatment was given by the health worker. Household surveys may be analyzed in conjunction with community health worker quality-of-care surveys, however, to provide information on correct assessment, classification, and treatment [16].

## Recommendations for Future Survey Protocols

The introduction of CCM provides a good example of the need for flexibility and continuous evolution in the major household surveys used to assess intervention coverage in low- and middle-income countries. Before 2005, few governments had authorized community health workers to provide treatment for childhood illnesses, and exceptions were limited to the provision of oral replacement salts for diarrhea. By 2011, 52 of the Countdown to 2015 priority countries had adopted CCM policies and had moved forward with implementation [4]. The widespread adoption of this and any new strategy, combined with a growing recognition of the need for evidence-based evaluations of program effectiveness, underline the continuing need for modifications in household survey protocols so that the population impact of specific strategies and interventions can be evaluated. We recommend strongly that all future coverage surveys include standard questions on the place and provider of treatment. Notably, when we shared our findings with technical staff at both DHS and MICS, they received our results positively and agreed to consider including questions capturing information on both place and provider of treatment in future surveys. The responsiveness of these survey programs to our suggestions should help to ensure the adequate capture of important changes in service delivery at a population level.

Importantly, additional questions incorporated into DHS and MICS surveys will need to be adapted to each country context. For example, it will be important to conduct a pretest to determine whether respondents are able to identify the community health worker trained to provide CCM in their area. Program implementers can contribute to the validity of such measurements by introducing strategies that will help child caregivers remember and report accurately about care received from a CCM-trained community health worker. In an assessment of village health workers delivering CCM in Bangladesh, for example, each worker was given a bright pink bag that was shown in pretests to be easy for mothers to remember, and to help them distinguish the CCM worker from other community-level workers [17]. Similar context-specific strategies to increase the salience and recall of an encounter with a community health worker trained in CCM are likely to increase the validity of place of treatment reports.

Those interested in evaluating CCM must also consider the potential role of informal providers in providing treatments for childhood illnesses, and must ensure that context-specific response options are included in the survey protocol to separate community health workers and informal providers at the community level. Finally, globally, more studies are needed to explore care seeking. In particular, care seeking that involves the informal sector needs to be better studied, and the impact of such care-seeking behavior on CCM program needs to be investigated. Information from such studies, and consideration of the other recommendations we make above, will ensure that the questions included in future surveys are correctly designed to provide the information that evaluators of CCM and other strategies need.

**Key Points**

- Low-income countries are increasingly adopting community case management (CCM) as a strategy for increasing the coverage of appropriate treatments for childhood illnesses.
- CCM program managers need to evaluate the effectiveness of their programs through time trend analyses that investigate whether treatment is being received at the community level.
- Population-based household surveys such as the Demographic and Health Surveys (DHS) and Multiple Indicator Cluster Surveys (MICS) are currently the only available means of obtaining data on treatment coverage by source of treatment in low-income countries.
- In an assessment of nine DHS/MICS surveys in five countries that are currently implementing CCM, we found that, although data on care seeking and treatment coverage are available, there is limited information on treatment source.
- We recommend that treatment source questions be included in future survey protocols, a recommendation that technical staff at DHS and MICS are now considering; we also recommend alternative analysis plans that implementers and evaluators may use to assess CCM programs.

## Supporting Information

Table S1 **Summary of illness care seeking, treatment, and place of treatment available by survey.** Details of questions used to measure care seeking, treatment, and place of treatment by country-specific survey and the core questionnaires.
(DOCX)

## Abbreviations

CCM: community case management
DHS: Demographic and Health Survey(s)
MICS: Multiple Indicator Cluster Survey(s)
