# Supplementary material for: Measuring Coverage in MNCH: Evaluation of Community-Based Treatment of Childhood Illnesses through Household Surveys
Source: PLoS Med. 2013 May 7;10(5):e1001384. doi: 10.1371/journal.pmed.1001384 (PMC3646213; doi:10.1371/journal.pmed.1001384)
Supplement: Table S1 — Summary of illness care seeking, treatment, and place of treatment available by survey. Details of questions used to measure care seeking, treatment, and place of treatment by country-specific survey and the core questionnaires. (DOCX) [file pmed.1001384.s001.docx]

**Table S1: Summary of illness careseeking, treatment and place of treatment available by survey**

**1.1: DHS survey table summaries**

| Ethiopia DHS 2005 | Child Illness Definition | Careseeking | Site of careseeking  (Public & Private Included) | Treatment |
| --- | --- | --- | --- | --- |
| Diarrhea | Has (NAME) had diarrhea in the last 2 weeks? (q515) | Did you seek advice or treatment for the diarrhea from any source? (q519)  Where did you seek advice or treatment? Anywhere Else? Recorded all places mentioned (q520)  *If 2 or more places from q520 circled:* Where did you first seek advice or treatment? (q522)  How many days after the diarrhea began did you first seek advice or treatment for (NAME)? (q523) | Govt Hospital/Clinic, Govt health center, Govt health post, Community Health Agent, NGO Health Facility, Private Hospital/Clinic/Doctor, Pharmacy, Drug Vendor, Shop, Traditional Healer, Other (q520) | Was he/she given any of the following to drink at any times since he/she started having the diarrhea: (a)A fluid made from an ORS packet like LEMLEM; (b) homemade sugar and salt solution; (c) other homemade fluid (q525)  Was anything (else) given to treat the diarrhea?(q526) *Recorded all treatments given, zinc and other remedies included* (q527)  *If given zinc,* how many times was (NAME) given zinc? (q529)  *Note: Questions on increased fluids and continued feeding included (q517-q518)* |
| Pneumonia | Has (NAME) had an illness with a cough at any time in the last 2 weeks? (q531)  When (NAME) had an illness with a cough, did he/she breathe faster than usual with short, rapid breaths or have difficulty breathing? (q532)  When (NAME) had this illness, did he/she have a problem in the chest or blocked or runny nose? (q533) | Did you seek advice or treatment for this illness from any source? (q537)  Where did you seek advice or treatment? Anywhere else? Recorded all sources mentioned (q538)  *If two or more sources circled,* where did you first seek treatment? (q540)  How many days after the illness began did you first seek advice or treatment for (NAME)? (q541) | Govt Hospital/Clinic, Govt health center, Govt health post, Community Health Agent, NGO Health Facility, Private Hospital/Clinic/Doctor, Pharmacy, Drug Vendor, Shop, Traditional Healer (q538) | At any time during the illness, did (NAME) take any drugs for the illness? (q543)  What drugs did (NAME) take? Any other drugs? Recorded all mentioned: *antibiotics listed as a response* (q544H-M)  Did you already have (NAME OF DRUG FROM 544) at home when the child became ill? Ask separately for each drug (A-M) given in 544 (q545)  *Note: Questions on increased fluids and continued feeding included (q535-q536)* |
| Fever | Has (NAME) been ill with a fever at any time in the last 2 weeks? (q530) | Did you seek advice or treatment for this illness from any source? (q537)  Where did you seek advice or treatment? Anywhere else? Record all sources mentioned (q538)  *If two or more sources circled,* where did you first seek treatment? (q540)  How many days after the illness began did you first seek advice or treatment for (NAME)? (q541) | Govt Hospital/Clinic, Govt health center, Govt health post, Community Health Agent, NGO Health Facility, Private Hospital/Clinic/Doctor, Pharmacy, Drug Vendor, Shop, Traditional Healer, Other (q538) | At any time during the illness, did (NAME) take any drugs for the illness? (q543)  What drugs did (NAME) take? Recorded all mentioned*: antimalarials listed as a response* (q544a-g)  Did you already have (NAME OF DRUG FROM 544) at home when the child became ill? Ask separately for each drug (A-M) given in 544 (q545)  *Note: Timing of treatment from illness onset and length of treatment by each antimalarial (q545A-545L). Questions on increased fluids and continued feeding included (q535-q536)* |
| Niger DHS 2006^1^ | **Child Illness Definition** | **Careseeking** | **Site of Careseeking**  **(Public & Private Included)** | **Treatment** |
| Diarrhea | Has (NAME) had diarrhea in the last two weeks? (q475) | Did you seek advice or a treatment for the diarrhea? (q481)  Where did you seek advice or treatment? Recorded all places mentioned (q482) | Hospital, Health Center, Health Post, Community Health Worker, Hospital/Private Clinic, Pharmacy, Private Doctor, Shop, Traditional healer, Mobile Vendor, Other (q482) | Did you give him/her one of the following things to drink? (a) a liquid prepared from an ORS packet (b) a homemade liquid recommended by the government (q478)  Was anything (else) given to treat the diarrhea? (q479)  If yes, what (else) was given to treat the diarrhea? *Recorded all treatments given, other remedies included* (q480)  *Notes: Questions on increased fluids and continued feeding included (q476-q477); zinc was not included as a treatment option.* |
| Pneumonia | Has (NAME) suffered from a cough at any time in the last 2 weeks? (q467)  If had cough, When (NAME) had the cough, did he/she breathe faster than normal with short and rapid breaths? (q468) | Did you seek advice or treatment for the fever/cough? (q470)  Where did you seek advice or treatment? Recorded all mentioned. (q471) | Hospital, Health center, Maternity, Family Health Center, Health Post, Hospital/Private clinic, “Cabinet/Salle de Soin,” Pharmacy, Mobile vendor, Shop, Traditional healer, Other (q471) | *No treatment questions* |
| Fever | Has (NAME) suffered from a fever at any time in the last 2 weeks? (q466) | Did you seek advice or treatment for the fever/cough? (q470)  Where did you seek advice or treatment? Recorded all mentioned. (q471) | Hospital, Health center, Maternity, Family Health Center, Health Post, Hospital/Private clinic, “Cabinet/Salle de Soin,” Pharmacy, Mobile clinic, Shop, Traditional healer, Other (q471) | Has (NAME) taken any medication for the fever? (q473)  What medication did (NAME) take? Recorded all mentioned, *includes antimalarials* (q474)  Did you already have (NAME OF DRUG FROM 474) at home or did you obtain it from another source? If more than one source given, ask where antimalarial was obtained the first time. *Possible responses listed are* “Home”; “Other source” or “Don’t know”. *Asked separately for each antimalarial (A-D) given in 474.* (q474E-q474Q)  *Note: Timing of treatment from illness onset and length of treatment by each antimalarial (q474C-545P)* |
| Mali DHS 2006^1^ | Child Illness Definition | Careseeking | **Site of Careseeking**  **(Public & Private Included)** | Treatment |
| Diarrhea | Has (NAME) had diarrhea in the last 2 weeks? (q475) | Did you seek advice or treatment for the diarrhea? (q481)  Where did you seek advice or treatment? (q482) | National Hospital, Regional Hospital, CSREF, CSCOM, Disp/Mater, Hospital/Private clinic, Community Health Worker Pharmacy, Private doctor, Other (q482) | Have you given him/her one of the following to drink? (a) a liquid prepared from an ORS packet (b) a homemade liquid recommended by the government (q478)  Was anything (else) given to treat the diarrhea? (q479)  If yes, what (else) was given to treat the diarrhea? *Recorded all treatments given, other remedies included* (q480)  *Notes: Questions on increased fluids and continued feeding included (q476-q477); zinc was not included as a treatment option.* |
| Pneumonia | Did (NAME) suffer from a cough at any time in the last 2 weeks? (q467)  If yes, when (NAME) suffered from a cough, did he/she breathe faster than normal with short and rapid breaths? (q468) | Did you seek advice or treatment for the fever/cough? (q470)  Where did you seek the advice or treatment? Recorded all mentioned. (q471) | National Hospital, Regional Hospital, CSREF, CSCOM, Disp/Mater, Hospital/Private Clinic, Cabinet de Soin, Community Health Worker, Mobile Vendor, shop, Traditional healer, Other (q471) | *No treatment questions* |
| Fever | Did (NAME) suffer from a fever at any time in the last 2 weeks? (q466) | Did you seek advice or treatment for the fever/cough? (q470)  Where did you seek the advice or treatment? Recorded all mentioned. (q471) | National Hospital, Regional Hospital, CSREF, CSCOM, Disp/Mater, Hospital/Private Clinic, Cabinet de Soin, Community Health Worker, Mobile Vendor, shop, Traditional healer, Other (q471) | Has (NAME) taken medications for the fever? (q473)  What medication did (NAME) take? Recorded all mentioned, *includes antimalarials*. (q474)  Did you already have (NAME OF DRUG FROM 474) at home or did you obtain it from another source? If more than one source given, ask where antimalarial was obtained the first time. *Possible responses listed are* “Home”; “Other source” or “Don’t know”. *Asked separately for each antimalarial (A-D) given in 474.* (q474E-q474Q)  *Note: Timing of treatment from illness onset and length of treatment by each antimalarial (q474C-545P)* |
| Ghana 2008 DHS | **Child Illness Definition** | **Careseeking** | **Site of careseeking**  **(Public & private included)** | **Treatment** |
| Diarrhea | Has (NAME) had diarrhea in the last 2 weeks? (q518) | Did you seek advice or treatment for the diarrhea from any source? (q522)  Where did you seek advice or treatment? Circle the appropriate code(s) (q523)  *If two or more places from q523 circled:* Where did you first seek advice or treatment? q525)  How many days after the diarrhea began did you first seek advice or treatment for (NAME)? (q526) | Govt hospital/polyclinic, Govt health center, Govt health post/CHPS, public mobile clinic, public fieldworker, Pvt. Hospital/clinic, pharmacy, Pvt. doctor, Pvt mobile clinic, Pvt. Fieldworker, PFG/PPAG clinic, maternity home, shop/market, traditional practitioner, drug peddler, other  (q523) | Was he/she given any of the following to drink at any time since he/she started having the diarrhea:(A) a fluid made from a special ORS sachet; (b) government-recommended home fluid (q528)  Was anything (else) given to treat the diarrhea? (q529) What else was given to treat the diarrhea? Anything else? *Record all treatments given, zinc and other remedies included* (q530)  *Note: Questions on increased fluids and continued feeding included (q520-521)* |
| Pneumonia | Has (NAME) had an illness with a cough at any time in the last 2 weeks? (q534)  When (NAME) had an illness with a cough, did he/she breathe faster than usual with short, rapid breaths or have difficulty breathing? (q535)  Was the fast or difficult breathing due to a problem in the chest or to a blocked or runny nose? (q536) | Did you seek advice or treatment for the illness from any source? (q540)  Where did you seek advice or treatment? Circle the appropriate code(s) (q541)  *If 2 or more places from q540 circled:* where did you first seek advice or treatment? (q543)  How many days after the illness began did you first seek advice or treatment for (NAME)? (q544) | Govt hospital/polyclinic, Govt health center, Govt health post/CHPS, public mobile clinic, public fieldworker, Pvt. Hospital/clinic, pharmacy, Pvt. doctor, Pvt mobile clinic, Pvt. Fieldworker, PFG/PPAG clinic, maternity home, shop/market, traditional practitioner, drug peddler, other (q541) | At any time during the illness, did  (NAME) take any drugs for the illness? (q546)  What drugs did (NAME) take? Any other drugs? Record all mentioned, *antibiotics listed as a response.* (q547)  Did you already have (NAME OF DRUG FROM 544) at home when the child became ill? Asked separately for each drug (A-I) given in 547 (q549)  *Note: Questions on increased fluids and continued feeding included (q538-539)* |
| Fever | Has (NAME) been ill with a fever at any time in the last 2 weeks? (q533) | Did you seek advice or treatment for the illness from any source? (q540)  Where did you seek advice or treatment? Circle the appropriate code(s) (q541)  *If 2 or more places from q540 circled:* where did you first seek advice or treatment? (q543)  How many days after the illness began did you first seek advice or treatment for (NAME)? (q544) | Govt hospital/polyclinic, Govt health center, Govt health post/CHPS, public mobile clinic, public fieldworker, Pvt. Hospital/clinic, pharmacy, Pvt. doctor, Pvt mobile clinic, Pvt. Fieldworker, PFG/PPAG clinic, maternity home, shop/market, traditional practitioner, drug peddler, other (q541) | At any time during the illness, did  (NAME) take any drugs for the illness? (q546)  What drugs did (NAME) take? Any other drugs? Record all mentioned, *antimalarials listed as a response* (q547)  Did you already have (NAME OF DRUG FROM 544) at home when the child became ill? Asked separately for each drug (A-I) given in 547 (q549)  *Note: Timing of treatment from illness onset and length of treatment by each antimalarial (q550-572). Questions on increased fluids and continued feeding included (q538-539)* |
| Malawi 2010 DHS | **Child Illness Definition** | **Careseeking** | **Site of careseeking**  **(Public & private included)** | **Treatment** |
| Diarrhea | Has (NAME) had diarrhea in the last 2 weeks? (q518) | Did you seek advice or treatment for the diarrhea from any source? (q522)  Where did you seek advice or treatment? Anywhere else? (q523)  *If two or more places recorded,* Where did you first seek advice or treatment? (q525)  How many days after the diarrhea began did you first seek advice or treatment for (NAME)? (q526) | Govt Hospital, Govt Health Center, Govt Health Post/Outreach, Govt Mobile clinic, Govt HSA, CHAM Hospital, CHAM Health Center, Pvt Hospital/Clinic, Pharmacy, Pvt doctor, Pvt Mobile clinic, Pvt HSA, BLM, MACRO, Youth Drop-in Centre, Shop, Traditional Practitioner, Other (q523) | Was he/she given a fluid made from a special packet called THANZI or ORS? (q528)  Was anything (else) given to treat the diarrhea? (q529)  What (else) was given to treat the diarrhea? Anything else? *Zinc and other treatments included as options.* (q530)  *Note: questions on increased fluids and continued feeding included (q520-521)* |
| Pneumonia | Has (NAME) had an illness with a cough at any time in the last 2 weeks? (q534)  If yes, When (NAME) had an illness with a cough, did he/she breathe faster than usual with short, rapid breaths or have difficulty breathing? (q535)  Was the fast or difficult breathing due to a problem in the chest or to a blocked or runny nose? (q536) | Did you seek advice or treatment for the illness from any source? (q540)  Where did you seek advice or treatment? Anywhere else? (q541)  *If two or more places recorded,* Where did you first seek advice or treatment? (q543)  How many days after the illness began did you first seek advice or treatment for (NAME)? (q544) | Govt Hospital, Govt Health Center, Govt Health Post/Outreach, Govt Mobile clinic, Govt HSA, CHAM Hospital, CHAM Health Center, Pvt Hospital/Clinic, Pharmacy, Pvt doctor, Pvt Mobile clinic, Pvt HSA, BLM, MACRO, Youth Drop-in Centre, Shop, Traditional Practitioner, Other (q541) | At any time during the illness, did (NAME) take any drugs for the illness? (q546)  What drugs did (NAME) take? *Record all mentioned. Drugs listed include antibiotic pill/syrup or injection and others* (q547);  *Questions on increased fluids and continued feeding included (q538-539)* |
| Fever | Has (NAME) been ill with a fever at any time in the last 2 weeks? (q533) | Did you seek advice or treatment for the illness from any source? (q540)  Where did you seek advice or treatment? Anywhere else? (q541)  *If two or more places recorded,* Where did you first seek advice or treatment? (q543)  How many days after the illness began did you first seek advice or treatment for (NAME)? (q544) | Govt Hospital, Govt Health Center, Govt Health Post/Outreach, Govt Mobile clinic, Govt HSA, CHAM Hospital, CHAM Health Center, Pvt Hospital/Clinic, Pharmacy, Pvt doctor, Pvt Mobile clinic, Pvt HSA, BLM, MACRO, Youth Drop-in Centre, Shop, Traditional Practitioner, Other (q541) | At any time during the illness, did (NAME) take any drugs for the illness? (q546)  What drugs did (NAME) take? *Record all mentioned. Drugs listed include antimalarials and others* (q547)  *Note: Timing of treatment from illness onset and length of treatment by each antimalarial (q552-571); Questions on increased fluids and continued feeding included (q538-539)* |
| Ethiopia 2011 DHS | **Child Illness Definition** | **Careseeking** | **Site of careseeking**  **(Public & private included)** | **Treatment** |
| Diarrhea | Has (NAME) had diarrhea in the last 2 weeks? (q514) | Did you seek advice or treatment for the diarrhea from any source? (q518)  Where did you seek advice or treatment? Anywhere else? (q519)  *If two or more places recorded,* Where did you first seek advice or treatment? (q521) | Govt Hospital, Govt health center, Govt Health station/clinic, Govt Health Post/HEW, NGO health facility, NGO VCHW, Pvt hospital, Pvt clinic, pharmacy, drug vendor/store, shop, traditional healer, other (q519) | Was he/she given any of the following to drink at any time since he/she started having the diarrhea: (q522)   1. A fluid made from a special   ORS packet like LEMLEM?   1. A government-recommended homemade fluid?   Was anything (else) given to treat the diarrhea? (q523) *Zinc and other treatments included as options.* (q524)  *Note: questions on increased fluids and continued feeding included (q516-517)* |
| Pneumonia | Has (NAME) had an illness with a cough at any time in the last 2 weeks? (q527)  When (NAME) had an illness with a cough, did he/she breathe faster than usual with short, rapid breaths or have difficulty breathing? (q528)  Was the fast or difficult breathing due to a problem in the chest or to a blocked or runny nose? (q529) | Did you seek advice or treatment for the illness from any source? (q533)  *If two or more places recorded,* Where did you first seek advice or treatment? (q536) | Govt Hospital, Govt health center, Govt Health station/clinic, Govt Health Post/HEW, NGO health facility, NGO VCHW, Pvt hospital, Pvt clinic, pharmacy, drug vendor/store, shop, traditional healer, other (q534) | At any time during the illness, did  (NAME) take any drugs for the illness?(q537)  What drugs did (NAME) take? *Record all mentioned. Drugs listed include antibiotic pill/syrup or injection and others* (q538);  *Questions on increased fluids and continued feeding included (q531-532)* |
| Fever | Has (NAME) been ill with a fever at any time in the last 2 weeks? (q525) | Did you seek advice or treatment for the illness from any source? (q533)  *If two or more places recorded,* Where did you first seek advice or treatment? (q536) | Govt Hospital, Govt health center, Govt Health station/clinic, Govt Health Post/HEW, NGO health facility, NGO VCHW, Pvt hospital, Pvt clinic, pharmacy, drug vendor/store, shop, traditional healer, other (q534) | At any time during the illness, did  (NAME) take any drugs for the illness?(q537)  What drugs did (NAME) take? *Record all mentioned. Drugs listed include antimalarials and others* (q538)  *Note: Timing of treatment from illness onset and length of treatment by each antimalarial (q539-552)* |
| DHS Core Phase 6 | **Child Illness Definition** | **Careseeking** | **Site of careseeking**  **(Public & private included)** | **Treatment** |
| Diarrhea | Has (NAME) had diarrhea in the last 2 weeks? (q514) | Did you seek advice or treatment for the diarrhea from any source? (q518)  Where did you seek advice or treatment? Anywhere else? (q519)  *If two or more places recorded,* Where did you first seek advice or treatment? (q521) | Govt Hospital, Govt health center, Govt health post, mobile clinic, fieldworker, Pvt hospital/clinic, pharmacy, Pvt doctor, Pvt mobile clinic, Pvt fieldworker, shop, traditional practitioner, market, other (q519) | Was he/she given any of the following to drink at any time since he/she started having the diarrhea: (q522)   1. A fluid made from a special   packet called [LOCAL NAME  FOR ORS PACKET]?   1. A pre-packaged ORS liquid? 2. A government-recommended homemade fluid?   Was anything (else) given to treat the diarrhea? (q523) *Zinc and other treatments included as options.* (q524)  *Note: questions on increased fluids and continued feeding included (q516-517)* |
| Pneumonia | Has (NAME) had an illness with a cough at any time in the last 2 weeks? (q527)  When (NAME) had an illness with a cough, did he/she breathe faster than usual with short, rapid breaths or have difficulty breathing? (q528)  Was the fast or difficult breathing due to a problem in the chest or to a blocked or runny nose? (q529) | Did you seek advice or treatment for the illness from any source? (q533)  *If two or more places recorded,* Where did you first seek advice or treatment? (q536) | Govt Hospital, Govt health center, Govt health post, mobile clinic, fieldworker, Pvt hospital/clinic, pharmacy, Pvt doctor, Pvt mobile clinic, Pvt fieldworker, shop, traditional practitioner, market, other | At any time during the illness, did  (NAME) take any drugs for the illness?(q537)  What drugs did (NAME) take? *Record all mentioned. Drugs listed include antibiotic pill/syrup or injection and others* (q538);  *Questions on increased fluids and continued feeding included (q531-532)* |
| Fever | Has (NAME) been ill with a fever at any time in the last 2 weeks? (q525) | Did you seek advice or treatment for the illness from any source? (q533)  *If two or more places recorded,* Where did you first seek advice or treatment? (q536) | Govt Hospital, Govt health center, Govt health post, mobile clinic, fieldworker, Pvt hospital/clinic, pharmacy, Pvt doctor, Pvt mobile clinic, Pvt fieldworker, shop, traditional practitioner, market, other | At any time during the illness, did  (NAME) take any drugs for the illness?(q537)  What drugs did (NAME) take? *Record all mentioned. Drugs listed include antimalarials and others* (q538)  *Note: Timing of treatment from illness onset and length of treatment by each antimalarial (q539-552); Questions on increased fluids and continued feeding included (q531-532)* |

(1) Translated into English by the review team**1.2: MICS survey table summaries**

| Ghana 2006 MICS | Child Illness Definition | Careseeking | Site of careseeking  (Public & private included) | Treatment |
| --- | --- | --- | --- | --- |
| Diarrhea | Has (name) had diarrhoea in the last two weeks, that is, since (day of the week) of the week before last? (CA1) | *No careseeking questions* | *N/A* | During this last episode of diarrhoea, did (name) drink any of the following: (A) A fluid made from a special packet called (ors)?; (B) Government-recommended homemade fluid (sugar-salt solution)? (CA2a & CA2b)  Where did you get the (ors packet from CA2A)? (CA4B) *Possible responses listed:*  Govt. Hospital/polyclinic, Govt. Health centre, Govt. Health post, Village health worker, Mobile/outreach clinic, Pvt hospital/clinic, Pvt physician, Pvt pharmacy, Pvt. Mobile clinic, Relative or friend, Shop, Traditional practitioner, Other  *Notes: Questions on increased fluids and continued feeding included (CA3, CA4); No zinc and no other treatment listed* |
| Pneumonia | Has (name) had an illness with a cough at any time in the last two weeks, that is, since (day of the week) of the week before last?(CA5)  When (name) had an illness with a cough, did he/she breathe faster than usual with short, quick breaths or have difficulty breathing? (CA6)  Were the symptoms due to a problem in the chest or a blocked nose?(CA7)  *Note: If rapid breathing was due to a “blocked nose” or “other”, no questions on treatment or careseeking were asked.* | Did you seek advice or treatment for the illness outside the home?(CA8)  From where did you seek care? Anywhere else? Circle all providers mentioned but do NOT prompt with any suggestions (CA9) | Govt. hospital/polyclinic, Govt. health centre, Govt. health post, Village health worker, Mobile/outreach clinic, Pvt hospital/clinic, Pvt physician, Pvt pharmacy, Pvt. Mobile clinic, Relative or friend, Chemical shop, Traditional practitioner, Drug peddlers  (CA9) | Was (name) given medicine to treat this illness?(CA10)  What medicine was (name) given? Circle all medicines given, *antibiotics and others listed as a response.* (CA11)  Where did you get the antibiotic? (CA11B) *Responses include:*  Govt. hospital/polyclinic, Govt. health centre, Govt. health post, Village health worker/, Mobile/outreach clinic, Pvt hospital/clinic, Pvt physician, Pvt pharmacy, Pvt Mobile clinic, Relative or friend, Chemical shop, Traditional practitioner, Drug peddlers |
| Fever | In the last two weeks, that is, since (day of the week) of the week before last, has (name) been ill with a fever?(ML1) | Was (name) seen at a health facility during this illness? (ML2) | *No specific sites of careseeking are asked. Only if the child was taken to a health facility.* | Did (name) take a medicine for fever or malaria that was provided or prescribed at the health facility?(ML3)  What medicine did (name) take that was provided or prescribed at the health facility? Circle all medicines mentioned, *antimalarials and others included*. (ML4)  Was (name) given medicine for the fever or malaria before being taken to the health facility? (ML5)  Was (name) given medicine for fever or malaria during this illness?(ML6)  What medicine was (name) given? Circle all medicines given, *antimalarials and others included*. (ML7)  How long after the fever started did (name) first take (name of anti-malarial from ML4 or ML7)? If multiple anti-malarials mentioned in ML4 or ML7, name all anti-malarial medicines mentioned. (ML9)  Where did you get the (name of anti-malarial from ML4 or ML7)? If more than one anti-malarial is mentioned in ML4 or ML7, refer to the first anti-malarial given for the fever (the anti-malarial given on the day recorded in ML9). (ML9a) *Responses include:*  Govt. hospital, Govt. health centre, Govt. health post, Village health worker, Mobile/outreach clinic, Pvt hospital/clinic, Pvt physician, Pvt pharmacy, Pvt Mobile clinic, Relative or friend, Chemical shop, Traditional practitioner, Drug peddlers, Other |
| Malawi 2006 MICS | **Child Illness Definition** | **Careseeking** | **Site of careseeking**  **(Public & Private included)** | **Treatment** |
| Diarrhea | Has (NAME) had diarrhea in the last two weeks? (CA1) | *No careseeking questions* | N/A | During the last episode of diarrhea, did (NAME) drink any of the following: (a) A fluid made from a special packet called Thanzi/ORS packet solution (b) Government recommended homemade fluid: fresh juice, tea, porridge, thobwa (CA2, CA2A-E, CA3)  *Note: zinc not included as treatment option; Questions on increased fluids and continued feeding included (CA4-CA5)* |
| Pneumonia | Has (NAME) had an illness with a cough at any time in the last 2 weeks? (CA6)  If yes, When (NAME) had an illness with a cough, did he/she breathe faster than usual with short, quick, breaths or have difficulty breathing? (CA7)  Where the symptoms due to a problem in the chest or blocked nose? (CA8)  *Note: If rapid breathing was due to a “blocked nose” or “other”, no questions on treatment or careseeking were asked.* | Did you seek advice or treatment for the illness outside the home? (CA9)  From where did you seek care? Anywhere else? (CA9) | Govt Hospital, Govt health centre, Govt heal post, Village health worker, Mobile/outreach clinic, Pvt hospital/clinic, Pvt physician, Pvt pharmacy, Pvt mobile clinic, Relative or Friend, shop, Traditional Practitioner, Other (CA9) | Was (NAME) given medicine to treat this illness? (CA10)  If yes, What medicine was (NAME) given? Circle all medicines given, *antibiotics and others included.* (CA11) |
| Fever | In the last 2 weeks, has (NAME) been ill with a fever? (ML1) | Was (NAME) taken to a health facility during his illness? (ML2) | *No specific sites of careseeking are asked. Only if the child was taken to a health facility*. | Did (NAME) take a medicine for fever or malaria that was provided or prescribed at the health facility? (ML3)  What medicine did (NAME) take that was provided or prescribed at the health facility? Recorded all mentioned. *Medicines listed included specific antimalarials.* (ML4)  Was (NAME) given medicine for the fever or malaria before being taken to the health facility? (ML5)  Was (NAME) given medicine for the fever or malaria during this illness? (ML6)  What medicine was (NAME) given? Circle all medicines given. *Medicines listed included specific antimalarials*. (ML7)  How long after the fever started did (Name) first take (name of anti-malarial from ML4 or ML7)? If multiple antimalarials mentioned in ML4 or ML7, name all anti-malarial medicines mentioned. Record the code for the day on which the first anti-malarial was given. (ML9) |
| Mali 2009 MICS^1^ | **Child Illness Definition** | **Careseeking** | **Site of careseeking**  **(Public & Private included)** | **Treatment** |
| Diarrhea | Has (NAME) had diarrhea in the last 2 weeks? (CA1) | *No careseeking questions* | *N/A* | During the course of diarrhea, was (NAME) given one of the following products to drink: (a) A liquid prepared from an ORS packet or “Keneyadji” (b) a homemade solution composed of salt, sugar, and water (c) another liquid recommended by the government. (CA4)  Was anything (else) given to treat the diarrhea? (CA5)  What (else) was given to treat the diarrhea? *Zinc and others included as treatment options.* (CA6)  *Note: Questions on increased fluids and continued feeding included (CA2-CA3)* |
| Pneumonia | In the last 2 weeks, was (NAME) sick with a cough? (CA7)  If yes, When (NAME) was sick with a cough, did he/she breathe faster than normal with fast and rapid breaths or did he/she has difficulty breathing? (CA8)  Was the difficulty with breathing due to a chest problem or blocked, runny nose? (CA9)  *Note: If rapid breathing was due to a “blocked nose” or “other”, no questions on treatment or careseeking were asked.* | Did you seek advice or treatment for any aspect of the illness? (CA10)  Where did you seek advice or treatment? Recorded all mentioned. (CA11) | Hospital, CSREF, CSCOM, Dispensary/Maternity, Pvt clinic/Medical Office, Cabinet de Soin, Pharmacy, Parent/Friend, Shop, Traditional healer, Mobile vendor, Other | Was (NAME) given any medication to treat this sickness? (CA12)  What medication was given to (NAME)? Recorded all mentioned. *List includes antibiotics.* (CA13) |
| Fever | At any time in the last 2 weeks, was (NAME) sick with a fever? (ML1) | Did you seek advice or treatment from somewhere or someone for the disease? (ML2)  From where? (ML3) | Hospital, CSref, Cscom, Dispensary/Maternity, Pvt clinic/Medical Office, Cabinet de soin, Salle de soin, Pharmacy, Parent/Friend, Shop, Traditional healer, Mobile vendor, Other (ML3A) | Was the child taken to a health facility during the illness? (ML4)  Did the health facility give the child medication for fever or malaria? (ML5)  What medication was given to (NAME)?, *Drugs listed included specific antimalarials* (ML6)  Was (NAME) given medication for the illness before going to the HF or at any point during the illness? (ML7-ML8), *Drugs listed included specific antimalarials* (ML9)  How long after onset of fever was antimalarial administered the first time? (ML11) |
| MICS4 Core | **Child Illness Definition** | **Careseeking** | **Site of careseeking**  **(Public & Private included)** | **Treatment** |
| Diarrhea | In the last two weeks, has (name) had diarrhoea? (CA1) | *No careseeking questions* | *N/A* | During the course of diarrhea, was (NAME) given one of the following products to drink: (a) A fluid made from a special packet called (local name for ORS packet solution)?; (b) A pre-packaged ORS fluid for diarrhoea?(c) (Government-recommended homemade fluid X)? (CA4)  Was anything (else) given to treat the diarrhea? (CA5)  What (else) was given to treat the diarrhea? *Zinc and others included as treatment options.* (CA6)  *Note: Questions on increased fluids and continued feeding included (CA2-CA3)* |
| Pneumonia | In the last 2 weeks, was (NAME) sick with a cough? (CA7)  If yes, When (NAME) was sick with a cough, did he/she breathe faster than normal with fast and rapid breaths or did he/she has difficulty breathing? (CA8)  Was the difficulty with breathing due to a chest problem or blocked, runny nose? (CA9)  *Note: If rapid breathing was due to a “blocked nose” or “other”, no questions on treatment or careseeking were asked.* | Did you seek any advice or treatment for the illness from any source? (CA10) | Govt hospital, govt health centre, govt health post, village health worker, mobile/outreach clinic, pvt hospital/clinic, pvt physician, pvt pharmacy, pvt mobile clinic, relative/friend, shop, traditional practitioner, other | Was (name) given any medicine to treat this illness? (CA12)  Was (NAME) given any medication to treat this illness? (CA12)  What medication was given to (NAME)? Circle all medicines given. *List includes antibiotics.* (CA13) |
| Fever | In the last two weeks, has (name) been ill with a fever at any time? (Ml1) | Was (name) taken to a health facility during this illness? (ML4) | *No specific sites of careseeking are asked. Only if the child was taken to a health facility*. | Was (name) taken to a health facility during this illness?(ML4)  Was (name) given any medicine for fever or malaria at the health facility?(ML5)  What medicine was (name) given?; Probe: Any other medicine? *Drugs listed included specific antimalarials* (ML6)  Was (name) given any medicine for the fever or malaria before being taken to the health facility?(ML7) Was (name) given any medicine for fever or malaria during this illness? (ML8), What medicine was (name) given?*Drugs listed included specific antimalarials* (ML9)  How long after the fever started did (name) first take (name of anti-malarial from ML6 or ML9)? (ML11) |

(1) Translated into English by the review team
